# CD133, CD15/SSEA-1, CD34 or side populations do not resume tumor-initiating properties of long-term cultured cancer stem cells from human malignant glio-neuronal tumors

**DOI:** 10.1186/1471-2407-10-66

**Published:** 2010-02-24

**Authors:** Cristina Patru, Luciana Romao, Pascale Varlet, Laure Coulombel, Eric Raponi, Josette Cadusseau, François Renault-Mihara, Cécile Thirant, Nadine Leonard, Alain Berhneim, Maria Mihalescu-Maingot, Jacques Haiech, Ivan Bièche, Vivaldo Moura-Neto, Catherine Daumas-Duport, Marie-Pierre Junier, Hervé Chneiweiss

**Affiliations:** 1 Glial Plasticity lab; Inserm UMR 894; University Paris Descartes; Paris, France; 2 Department of Anatomy, UFRJ, Rio, Brazil; 3 Department of Neuropathology, Sainte-Anne Hospital, Paris, France; 4 Grenoble - Institut de Neurosciences INSERM U83638706 La Tronche cedex, France; 5 Inserm U955, Team 10, University of Paris 12, Créteil, France; 6 Laboratoire de génomique cellulaire des cancers (FRE 2939 CNRS), Institut Gustave Roussy, Villejuif, France; 7 CNRS UMR 7175; Institut Gilbert Laustriat; University Strasbourg I; Illkirch, France; 8 Génétique et Biothérapies des Maladies Dégénératives et Prolifératives du Système Nerveux INSERM U745; IFR71; Université Paris Descartes; Paris, France; 9 Present address: Department of Neurosurgery, Annecy, France

## Abstract

**Background:**

Tumor initiating cells (TICs) provide a new paradigm for developing original therapeutic strategies.

**Methods:**

We screened for TICs in 47 human adult brain malignant tumors. Cells forming floating spheres in culture, and endowed with all of the features expected from tumor cells with stem-like properties were obtained from glioblastomas, medulloblastoma but not oligodendrogliomas.

**Results:**

A long-term self-renewal capacity was particularly observed for cells of malignant glio-neuronal tumors (MGNTs). Cell sorting, karyotyping and proteomic analysis demonstrated cell stability throughout prolonged passages. Xenografts of fewer than 500 cells in Nude mouse brains induced a progressively growing tumor. CD133, CD15/LeX/Ssea-1, CD34 expressions, or exclusion of Hoechst dye occurred in subsets of cells forming spheres, but was not predictive of their capacity to form secondary spheres or tumors, or to resist high doses of temozolomide.

**Conclusions:**

Our results further highlight the specificity of a subset of high-grade gliomas, MGNT. TICs derived from these tumors represent a new tool to screen for innovative therapies.

## Background

Tumor initiating cells (TICs) from various types of cancers have been isolated and characterized. The tumors of origin range from glioblastomas and medulloblastomas [1-6] to epithelial tumors of the breast [7], lung [8], colon [9], and prostate [10]. Gliomas represent the majority of primary tumors from the central nervous system (CNS) [11]. Difficulties in clinical management (e.g. treatment and prognosis) are related to the complex identity of gliomas, which lack reliable morphological and molecular signatures, precluding thus the establishment of a clear cut classification discriminating between different tumor subtypes [12].

Historically, it has been proposed that gliomas (astrocytomas and oligodendrogliomas) originate respectively from mature astrocytes or oligodendrocytes. The fact that these brain tumors frequently include a mixture of cells expressing neuronal and glial markers, has recently led to the alternative proposal that gliomas arise from neural stem/progenitor cells. Support for this hypothesis comes from mouse models in which changes in the expression of oncogenes or tumor suppressors lead to experimental tumors [13]. Neural progenitor cells are, for example, more sensitive than differentiated astrocytes to the oncogenic effects of combined over-activation of Ras and Akt signaling pathways [14]. It should however be kept in mind that glioblastomas, the most malignant form of gliomas, can be generated in mice by retroviral transduction of oncogenes into mature glial cells [14-16]. In good agreement, the conversion of mature astrocytes toward neural progenitors induced by TGFα [17], a growth factor overexpressed early in the development of human gliomas [18]sensitizes them to cancerous transformation [19]. The isolation from human glioblastoma biopsies of malignant cells that express markers of neural stem cells supports the existence of tumor stem cells within gliomas [1-3,6]. Most importantly, some of these cells exhibit the true properties of tumor initiating cells (TICs), including the ability to give rise to a tumor identical to the one observed in the patient upon orthotopic grafting in mouse brains [1,3,6]. It remains, however, unknown whether these TICs might help to discriminate between glioma sub-types. Moreover, the design of specific therapies awaits the identification of the molecular pathways presiding over the maintenance of the properties of these tumor stem cells.

Here, we sought for tumor stem-like cells in 47 human adult malignant glial tumors. We identified a subset of glial tumors that contain at high frequency of cells generating long-term self-renewing floating spheres in vitro, and novel tumors in immunodeficient mice. This subset corresponds to malignant glio-neuronal tumors (MGNTs) [20]. MGNTs are World Health Organization grade III and IV tumors that always present numerous glial fibrillary acidic protein (GFAP)- and a few neurofilament protein (NFP)-positive tumor cells. The other neuronal markers tested (NeuN, synaptophysin, and chromogranin) are inconstantly expressed. Distinction of MGNTs from other malignant gliomas is of clinical importance since gross total surgical resection of these tumors is the major prognostic factor predicting long-term survival [20]. Flow cytometry and 2D-SDS-PAGE analyses showed stable and common proteomic profiles of MGNT-derived tumor initiating cells growing as floating spheres. These cells are highly resistant to temozolomide and thus represent a novel tool to screen for more efficient therapies.

## Methods

### Sample Classification

All of the samples were classified according to World Health Organization guidelines (grade II, III or IV for gliomas), and the classification of Sainte Anne Hospital (low grade oligodendroglioma or type A, high grade oligodendroglioma or type B, glioblastomas and malignant glio-neuronal tumors, [20]. The biopsies were collected by a pathologist in the surgical room from July 2002 to July 2005. All patients were 18 years old or older, had signed a written agreement for participation to the research project after having being informed of the goals, potential interest of the research and methods. This biomedical research was conducted according to the declaration of Helsinki, to the French laws, and was approved by the institutional review board of Ste Anne Hospital, Paris. Anatomopathological diagnosis classified tumors as glioblastoma multiformis (n = 6), Malignant Glio-Neuronal Tumors (MGNTs, n = 15), medulloblastoma (n = 2), ganglioglioma (n = 1), oligodendroglioma (n = 23). Immunolabeling of formalin-fixed, paraffin-embedded tissue sections was performed as previously described [20].

### Cell cultures

Viable fragments were transferred to a beaker containing 0.25% trypsin in 0.1 mM EDTA (4:1), and slowly stirred at 37°C for 30-60 min. Dissociated cells were plated in 75 cm^2 ^tissue culture flasks plated at 2500-5000 cells/cm^2 ^in Dulbecco's modified Eagle's: F-12 medium (1:1) containing the N2, G5 (containing FGF and EGF) and B27 supplements (all from Invitrogen, France). After 2 to 47 days in culture, spheres bloomed from clusters of adherent cells. They were dissociated in single-cell suspension each week with a renewal of two third of their culture medium.

### Characterization of sphere-forming cells

Dissociated sphere-derived cells were plated in 18-mm diameter wells in 1 ml volumes at a density of 200 000 cells/well. Cell proliferation/viability was evaluated using the WST1 kit from Roche according to the manufacturer's protocol. We verified that the results were the same as those obtained by counting the absolute number of viable cells using trypan blue exclusion. Clonality was evaluated in 96-well plates seeded at a cell density of 1-200 cells/well/0.1 ml. The number of wells containing at least one secondary sphere was evaluated after 3-4 weeks of culture.

### Flow cytometry analysis of cell surface antigens

Single-cell suspensions were incubated with fluorochrome-conjugated monoclonal antibodies: fluorescein (FITC)-coupled CD15, FITC-CD11b, FITC-CD45, phycoerythrin (PE)-coupled CD34, PE-Thy-1 (CD90), PE-CD133 and phycoerythrin cyanin 5 (PC5)-coupled CD34, PC5-CD56, and allophycocyanin (APC)-coupled CD133. CD133 antibodies were from Miltenyi (France), all others from Beckman-Coulter. Dead cells were excluded from the analysis by 7-AAD. Acquisition was performed on a Facscalibur (Becton Dickinson) and viable cells were analyzed with Cellquest software.

### Assessment of the neural differentiation potential

Cells were plated at a density of 5.000 cells/cm^2 ^onto polyornithine-coated glass coverslips and onto Lab-Tek™ II Chamber Slide™ System (Nalge Nunc International) http://www.nuncbrand.com/page.aspx?ID=234 in the presence of FCS or B27 (Invitrogen) for 2 to 14 days. Multiple immunofluorescence assays for neural antigens were performed as previously described [17,20].

### Real-time RT-PCR

The theoretical and practical aspects of real-time quantitative RT-PCR using the ABI Prism 7900 Sequence Detection System (Perkin-Elmer Applied Biosystems) have been described in detail elsewhere [21]. Briefly, total RNA is reverse-transcribed before real-time PCR amplification. Quantitative values are obtained from the threshold cycle (Ct) number at which the increase in the signal associated with exponential growth of PCR products begins to be detected using PE Biosystems analysis software, according to the manufacturer's manuals. We also quantified transcripts of *TBP *gene, which encodes the TATA box-binding protein as the endogenous RNA control, and each sample was normalized on the basis of its TBP content.

Results, expressed as N-fold differences in target gene expression relative to the *TBP *gene, termed N*target*, were determined by the formula: N*target *= 2^ΔCt_*sample*_^, where ΔCt value of the sample was determined by subtracting the Ct value of the target gene from the Ct value of the *TBP *gene.

The N*target *values of the samples were subsequently normalized to a "basal mRNA level", i.e. normalized to the smallest amount of target gene mRNA detectable and quantifiable by real-time quantitative RT-PCR assays (target gene Ct value = 35; N*target *value = 1).

The nucleotide sequences of primers for *TBP *and the 4 target genes are available on request. The thermal cycling conditions comprised an initial denaturation step at 95°C for 10 min and 50 cycles at 95°C for 15 s and 65°C for 1 min. Experiments were performed with duplicates for each data point.

### Chromosome Analysis of neurosphere-derived cells

Cells were treated with medium containing 10 μg/ml Colchicine for 1 to 2 hours, and resuspended in hypotonic 1% sodium citrate at room temperature for 30 minutes. The cells were then washed in a methanol-acetic acid (3:1, v/v) fixative solution for 30 minutes, and spread onto clean dry slides. Q-banding staining was then performed, and 10 metaphases were analyzed for each sample.

### Proteomic analysis

Cells were washed three times with PBS for 10 min with gentle shaking, prior to lyses in buffer containing 8 M urea, 4% CHAPS and 40 mM Tris. The protein pellets were dissolved in isoelectric focusing buffer and quantified using the Micro BCA protein assay kit (Pierce). First-dimension isoelectric focusing was performed with the Ettan IPGphor system (Amersham Biosciences) at 20°C with a maximum current setting of 50 mA/strip. Immobiline DryStrip gels (IPG stripes, Amersham Biosciences) with a pH gradient of 3-10 or 4-7 and a length of 13 cm were rehydrated in 250 μl sample solution, containing 80 μg proteins. Prior to SDS-PAGE analysis, IPG stripes were equilibrated in second-dimension equilibration buffer (6 M urea, 2% SDS, 50 mM Tris-HCl, pH 8.8, 30% glycerol) containing 1% DTT (Sigma) to reduce the disulfide bonds of the proteins. The second dimension was carried out using 12.5% gradient polyacrylamide gels at constant 20 mA current per gel. Comparison of the protein maps was achieved using the PD-Quest software according to the manufacturer instructions (BioRad).

## Results

### Selection and expansion of cell-forming spheres from adult human brain tumors

In order to preserve tissue architecture and improve sample handling as well as the versatility of the tests that may be applied, we developed a two-step strategy for the 49 surgical samples of our survey. Tumor samples were first sliced in the surgical room in fragments smaller than 1 mm^3^, and placed on surgical sponges bathed with culture medium. Tumor fragments were either dissociated within 2 hours after surgery (28 out of 49, 58%), or incubated in organotypic conditions for a maximal time of 2 weeks prior dissociation. Tumor fragments cultured on surgical sponges are viable and conserve the tumor architecture for up to 6 weeks (additional file 1). In both cases, the dissociated cells were plated at low density (<5 × 10^3 ^cells/cm^2^) in a serum-free medium containing EGF and FGF2 (G5 supplement).

In twelve of the fifteen cultures of MGNTs, floating cellular spheres were observed after 14 to 60 days in culture, and could be amplified from 6 months up to 52 months. In contrast, none of the other 23 tumors classified as World Health Organization grade II or III oligodendrogliomas (*n *= 9 and 14, respectively) generated spheres up to 90 days, the time at which no viable cells remained in the cultures. Similarly, two benign lesions, one Taylor Dysplasia and one adult pilocytic astrocytoma, did not yield any sphere. A third in vitro behavior pattern was observed for cells derived from three of the six glioblastoma multiformis studied, two adult medulloblastomas and one adult ganglioglioma. In these cases, spheres developed but stopped dividing after 2 to 5 months of culture, and disappeared suggesting limited auto-renewal capabilities (Table 1).

**Table 1:** Summary of the brain tumor cultures. See text for details.

Tumor type	Number of cases	Number of cultures containing floating cellular spheres	Self-renewal length ≤ 6 months	Self-renewal length > 6 months
**MGNT**	15	12	0	12

**Oligodendroglioma II**	9	0	-	-

**Oligodendroglioma III**	14	0	-	-

**Glioblastoma**	6	3	3	0

**Pilocytic astrocytoma**	1	0	-	-

**Taylor Dysplasia**	1	0	-	-

**Ganglioglioma**	1	1	1	0

**Medulloblastoma**	2	2	2	0

These data extended previous reports of absence of EGF/FGF2-responsive clonogenic precursors in oligodendrogliomas [2-4,22]. They revealed the unique ability of MGNTs to yield cellular spheres that could be amplified for very long periods.

### MGNT contain cells endowed with long-term self-renewal and clonal properties

MGNT cells were moderately proliferative but highly clonogenic. The average proliferation rate, determined using cultures derived from MGNT 1, 6 and 7 (TG1, TG6 and TG7), was equal to 236% ± 27% within 48 hours. In two separate experiments, the percentage of cells initiating-spheres, determined by plating 500 dissociated cells in 6-well plates and assessing the number of secondary spheres, reached 25-30%. Similar results were obtained when cells were plated at a density of 200 cells per well in 96-well plates (TG1, 28.9% ± 8.0; TG6, 15.5% ± 7.0; TG7, 15.6% ± 5.0, mean ± sem, *n *= 3) (Table 2). Finally, 7 to 10% of the cells seeded at one cell/well generated at least one secondary sphere after one month in culture (TGI, 9 ± 3%, n = 4, TG6, 7 ± 1%, n = 3, TG7, 7 ± 2%, mean ± sem, n = 3). These properties were reproducibly observed for TG1-cells from week 40 to week 100, the latest time tested.

**Table 2:** Clonal properties of MGNT derived glioma stem cells

Culture name	200 cells/well	1 cell/well
**TG1**	28.9 ± 8	9 ± 3

**TG6**	15.5 ± 7.0	7 ± 1

**TG7**	15.6 ± 5.0	7 ± 2

Example of clonal properties of cell-initiating spheres derived from MGNT. The cellular spheres were dissociated, and plated at 200 or 1 cell/well in 96-well plates. The percentage of wells containing spheres is presented as mean ± sem, n = 3.

### MGNT-derived cells forming spheres exhibit a neural progenitor phenotype in vitro

**Figure 1:**
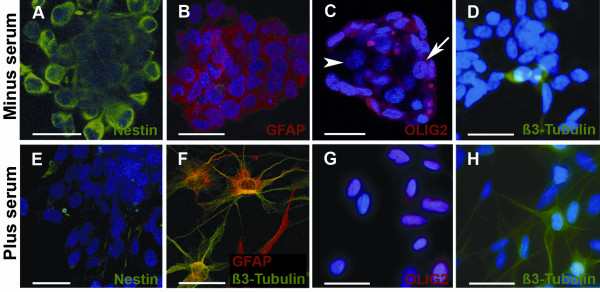
**Immunocytochemical characterization of cells derived from MGNT**. MGNT-derived cells form spheres in culture and express neural markers. Example of spheres from one tumor, TG1, cultured with FGF/EGF (**A-D**) or in the presence of 10% (**E-F**), or 0,5% FCS **(G)**. **A-D**. In serum-free medium, cells within spheres expressed nestin (green, **A**), GFAP (red, **B**), Olig2 (red, **C**) and low levels of ß3-tubulin (green, **D**). **E-G**. After 14 days in serum-containing medium, the numbers of cells expressing Nestin decreased (green, **E**), The differentiated cells retained nuclear Olig2 expression (red, **G**). Some cells exhibited co-immunolabelling for GFAP (red) and ß3-tubulin (green, **F**). **H**. ß3-tubulin-immunoreactive cells in serum-free medium supplemented with B27. Nuclei were counterstained with DAPI or Topro. Scale bar = 40 μm. **I**. Quantification of cells expressing nestin, GFAP, Olig2 and ß3-tubulin in serum-free medium containing EGF and bFGF, in medium supplemented with 0.5 and 10% FCS, and in medium supplemented with B27.

Analysis was conducted with a panel of antibodies directed against antigens characteristic of stem/progenitor cells of the CNS and other tissues, and of cells of the neuronal and glial lineages (Figure 1A-D). Numerous cells expressed nestin and the bHLH-transcription factor Olig2 (Figure 1A, 1C and 1I), normally expressed in stem/progenitor cells. A majority of cells were immunoreactive for GFAP, an astroglial marker (Figure 1B and 1I), whereas a small population was immunoreactive for the neuronal marker ß3-tubulin (Figure 1D and 1I). Each individual sphere exhibited cellular heterogeneity.

When bFGF and EGF were removed and replaced by serum, most but not all spheres underwent changes characteristic of differentiation: they adhered rapidly to the plastic substrate, the cells flattened and acquired a fusiform shape. Cellular heterogeneity both between spheres and within each sphere was maintained in these conditions, as shown by immunocytochemical analysis (Figure 1E-G and 1I). When treated with 0.5% FCS for 14 days, all cells exhibited nuclear Olig2-immunolabeling (Figure 1G and 1I). In the presence of B27, a cocktail designed to promote neuron survival and development in cultures of normal nervous tissue, 30% of the cells within the spheres acquired the appearance of neuron-like cells with a rich neurite-like branching, and ß3-tubulin expression (Figure 1H and 1I). Interestingly, a fraction of the differentiated cells co-expressed neuronal and glial markers as in the original tumor (Figure 1F and 1I).

### Cell surface phenotype and the functional state of sphere-derived cells

**Figure 2:**
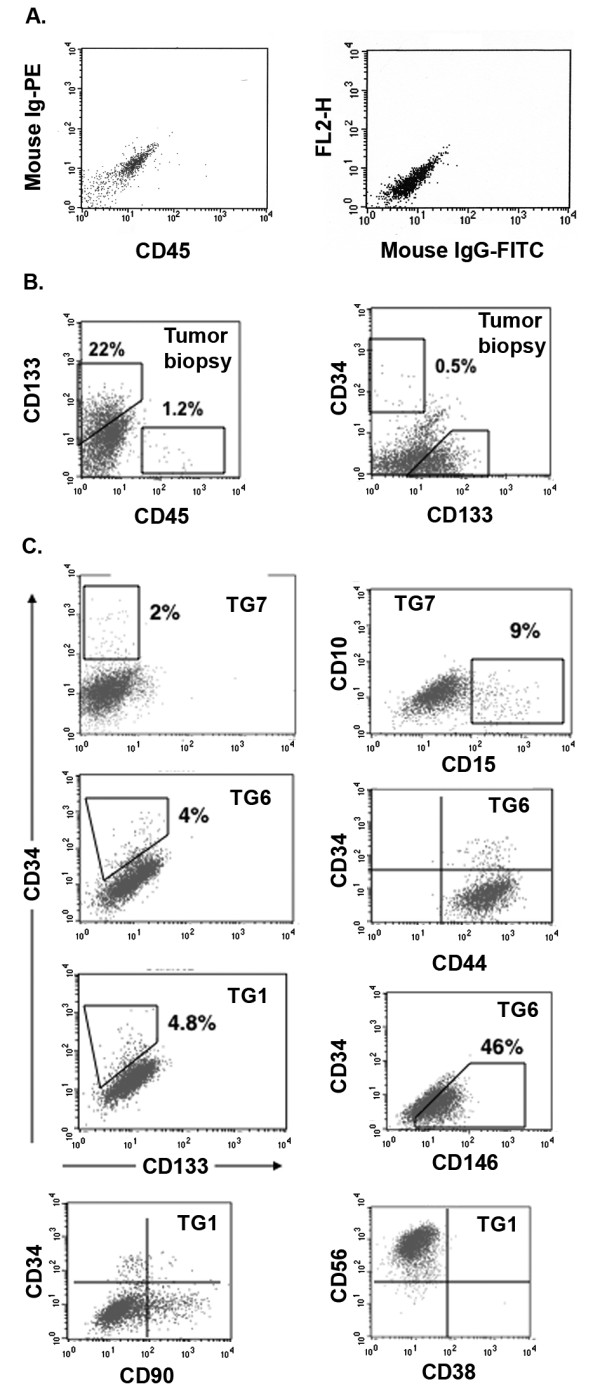
**Expression of surface antigens by from freshly dissociated MGNTs cells and MGNT-derived cells forming spheres in culture**. **A**. Control IgGs. **B**. Fresh tumors were dissociated enzymatically with trypsin and immediately immunolabeled with either CD133 (left panel) or CD34 (right panel). **C**. MGNT-derived cellular spheres were dissociated mechanically as described (see text), and immunolabeled. Cells were analyzed by flow cytometry and the positive gates indicated were defined based on analysis done after double labeling with FITC- or PE-labeled isotype-matched non-immune immunoglobulin and another specific antibody. Results are expressed in a morphological gate including all viable nucleated cells. Data shown are from one experiment out of three giving similar results.

The cell-surface phenotype of sphere-derived cells from different MGNT cultures (TG1, TG6, TG7) was repeatedly characterized using flow cytometry and remained stable over 6 months and up to 52 months for TG1, the latest time tested (Figure 2). CD56/NCAM, CXCR4, CD90/Thy-1, and VLA2, which are known to be express by neural progenitors, were detected in over 90% of the cells. A second group of proteins was detected in less than 10% of the cells. They included CD15/Lex/ssea1, described on neural stem cells [23], and CD34 synthesized by stem/progenitor cells from several tissues, but which so far has not been reported on neural cells. CD34 is known to be also expressed by endothelial cells, that may originate from neural stem cells in normal brains [24]. However, no expression was detected for the endothelial-specific markers CD31 and VE-cadherin by MGNT-derived cultured cells. Similarly, none of the hematopoietic markers CD45, CD3, CD11b, CD14, CD19, CD33, CD36, CD38 was expressed, thus excluding that CD34 expression reflected contamination by endothelial or hematopoietic progenitors. Likewise, we did not detect expression of the cytokine receptors c-kit or Flt3, or CD44 and CD93. In addition, we looked for CD133-immunoreactive cells that have been reported to represent up to 50% of dissociated tumor cells examined by flow cytometry [1-6]. In agreement with these data, we found that 20 to 30% of cells isolated immediately after tumor resection were positive for this antigen (n = 4). Surprisingly, only a few cultured cells expressed CD133, suggesting a rapid down-regulation in vitro. Furthermore, CD133+ as well as CD133- cells were able to form floating spheres as well as to initiate tumors upon brain grafting (see below). One may hypothesize that CD133 expression is not a characteristic of a subpopulation of cells endowed with stem-like properties at least in MGNTs, but rather depends on their environment.

To determine if the sphere-forming capability segregated according to CD34 or CD15 expression, we sorted the negative and positive fractions from TG1, and TG6 cultures (week 35 to 90), and determined their ability to generate spheres in culture. The ability to form spheres segregated neither with CD15 nor with CD34 expression (Table 3). A side population was identified using the Hoechst 3342 dye. Cells excluding the dye amounted to 2-10% of the cellular population (2 independent experiments). The capacity of the cells to form spheres (in both primary and secondary assays) was independent of their ability to exclude the dye, suggesting that this property depends from the cell activation state rather than its identity.

**Table 3:** Phenotypic characterization of cell-forming spheres.

	% of wells containing spheres	Number of sphere/well
**CD15-**	11/11 = 100%	15 ± 2.2 (day 21)

**CD15+**	9/13 = 70%	5.9 ± 2.6 (day 21)

**CD34-**	14/14 = 100%	22 ± 4.1 (day 36)

**CD34+**	12/15 = 80%	20 ± 3.6 (day 36)

**Non SP**	10/10 = 100%	18.6 ± 0.5 (day 36)

**SP**	10/10 = 100%	19.1 ± 0.2 (day 36)

Cells cultured as spheres from either TG1, TG6 or TG7 were dissociated and sorted according to their expression of CD15, CD34 or they capability to exclude Hoechst 3342 dye. Sorted cells were then plated (100 cells/well) in round-bottomed 96-well plates and fed once a week. Wells were examined at days 21 and 36, and the number of spheres (with more than 50 cells) per well was recorded. Data are from 1 representative experiment out of 3.

RT-PCR analysis was additionally used to seek for transcripts usually observed in stem cells and considered as involved in their self-renewal capacities. Oct 4, Nanog, and hTERT transcripts were observed in all MGNT-derived cells forming spheres analyzed (n = 3) (Table 4). Of note, CD133 transcripts were always close to the limit of detection (Table 4).

**Table 4:** Expression of stemness markers by MGNT-derived cells forming spheres.

Samples	*OCT4/POU5F1*	*NANOG*	*HTERT*	*CD133/PROM1*
**TG1**	5937 ^a^	1240	131	5

**TG6**	11857	1959	165	12

**TG10**	1686	169	517	3

**TG16**	871	611	73	14

*OCT4/POU5F1, NANOG, HTERT *and *CD133/PROM1 *genes expressions were evaluated using real-time quantitative PCR in 4 MGNT-derived cells forming spheres. Tests were done with cultures at passage 30 ± 5. Data are from one experiment out of three giving similar results. ^a ^N-fold differences in target gene expression relative to the smallest amount of target gene mRNA detectable and quantifiable by real-time quantitative RT-PCR assays (target gene Ct value = 35; Ntarget value = 1).

**Figure 3:**
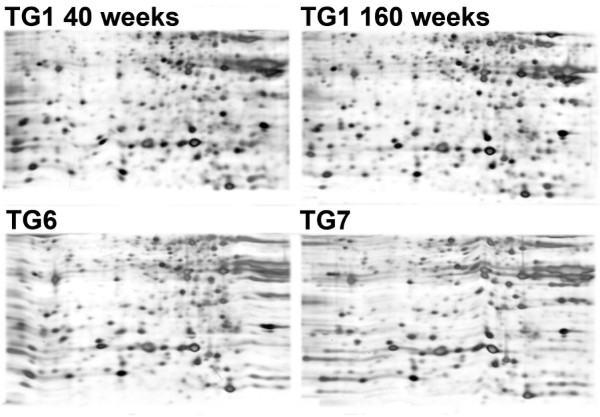
**Proteomic patterns of MGNT-derived cells forming spheres**. Upper panel: two-dimensional gel electrophoresis analysis for TG1, one of the cultures of MGNT-derived cells forming spheres, TG1, at 40 (left) and 160 (right) weeks of culture. Lower panel: the same area of the gel as in the upper panels for protein extracts taken from two other cultures of MGNT-derived cells forming spheres, TG6 and TG7.

Molecular characterization of cells-forming spheres was further performed using 2D-SDS-PAGE. The stability of the proteomic profile of a given culture was determined using TG1 cells at two different passages (week 40 and week 160). The proteomic profiles of cells isolated from the spheres were also compared between different MGNTs. Analysis after silver staining revealed a very similar protein pattern for TG1 at both passages examined, as well as between cells derived from distinct patients (TG6, TG7) (Figure 3). Comparison of the protein maps using computer-assisted analysis (PD-Quest software) showed a high coefficient of correlation (0,84 ± 0.06). A differential subtractive approach comparing protein patterns from cells collected from MGNT-derived spheres and a frozen micro-dissected fragment of the original tumor, allowed the MALDI-TOF identification of a group of proteins enriched in cultured cells (Table 5). The eight potential biomarkers identified included the mannose-6-phosphate receptor binding protein 1 reported on early stem cells [25], and two proteins associated with multidrug resistance, Sorcin being the most strikingly over-expressed. Sorcin mRNA and protein levels have been reported in astrocytoma and are positively correlated with the malignancy of the tumor [26]. These data are consistent with the possibility that Sorcin expression signs for an enhanced occurrence of TICs in high-grade gliomas.

**Table 5:** MALDI MS/MS characterization of proteins overexpressed in MGNT-derived cells forming spheres.

Entry SwissProt	Masse	Pi	% cover	Identification
RET1_HUMAN	15879.92	4,99	60	(P09455) Retinol-binding protein I

SORCN_HUMAN	21947.46	5,32	30	(P30626) Sorcin (22 kDa protein) (CP-22) (V19)

GSTP1_HUMAN	23438.07	5,44	27	(P09211) Glutathione S-transferase P (EC 2.5.1.18)

PRDX6_HUMAN	25002.19	6,02	25	(P30041) Peroxiredoxin 6 (EC 1.11.1.15)

ECHM_HUMAN	31823.30	8,34	29	(P30084) Enoyl-CoA hydratase

HSPB1_HUMAN	22825.51	5,98	66	(P04792) Heat-shock protein beta-1 (HspB1)

M6PBP_HUMAN	47189.00	5,30	53	(O60664) Mannose-6-phosphate receptor binding protein 1

Q96E67_HUMAN	40542.00	5,55	32	(Q96E67) ACTB protein (Fragment)

The proteins listed in the table were only detected in 2D-SDS silver stained gels obtained from MGNT-derived cells forming spheres and not observed in the corresponding gel obtained with the same amount of proteins extracted from the original brain tumor. The proteins were identified by mass spectrometry analysis.

Altogether, these results demonstrate that cells forming cells derived from MGNT exhibit a common and stable surface antigen and proteomic profile, as well as some features of stem/progenitor cells in long-term cultures.

### MGNT-derived spheres contain tumor-initiating cells

Karyotype analysis and in vivo behavior upon orthotopic grafting were performed in order to determine whether the cell-forming spheres derived from MGNTs were indeed tumoral and behaved as tumor-initiating cells (TICs).

**Figure 4:**
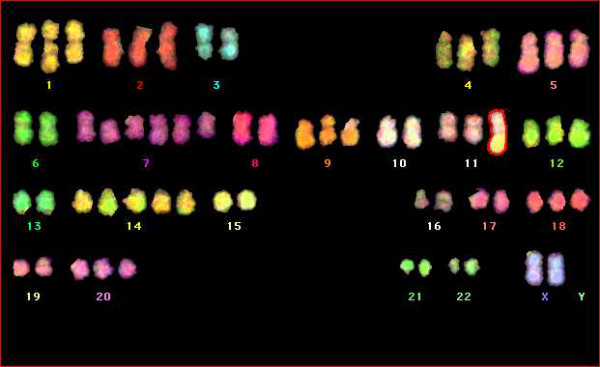
**Karyotype**. Chromosomal analysis of TG1 cells by Multi FISH reveals a hypotriploidy with an over-representation of chromosomes 7 and 14, and one chromosome 11q+.

Karyotype analysis was performed on cells collected from spheres grown from 4 tumors (TG1, TG6, TG7, TG10). Gains were observed for each cell examined on chromosomes 1q, 5, 7, 9q, 12, and 14, whereas losses were observed on chromosomes 9p and 18q (Fig 4).

**Figure 5:**
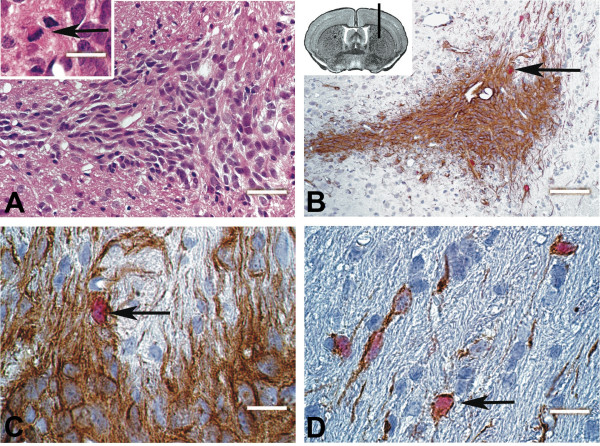
**Intra-cerebral grafts**. MGNT-derived cells forming spheres were grafted in the dorsal striatum of immunodeficient mice (inset in panel B). Mice were sacrificed from 12 to 22 weeks post-graft and brain sections immunostained. **A**. Hematoxylin-eosin staining was performed to identify tumor mass (inset: a cell undergoing mitosis within the tumor mass). **B**. Identification of human cells with an antibody specific of the human vimentin (brown). **C-D**. Double immunolabeling for vimentin (brown) and minichromosome maintenance 2 (mcm2, red nuclear labeling) identified cycling human cells (arrows). Cells were able to migrate at distance from the site of injection (D). Scale bar: 100 μm in B, 50 μm in A, 20 μm in C, D and inset in A. B-D: floxin counterstaining.

To determine the presence of TICs, MGNT-derived sphere cells from four different MGNTs were grafted into the brain of nude mice (2 mice each, 200-1000 living cells per graft). The recipients were sacrificed from 12 to 24 weeks post-graft. Tissue analysis after hematoxylin/eosin and immunolabeling with antibodies that recognized only the human form of the glial marker vimentin identified human cells in 7 out of 8 mice. In six cases out of seven, the grafts had developed with cell proliferation (as evidenced by Mcm2 labeling), and migration over large distances from the injection site (Fig 5). Several migrating cells were observed along the neural fiber axis (Fig 5).

Taken altogether these data demonstrate that long-term cultured MGNT-derived floating spheres contain tumor cells with neural stem/progenitor phenotype, thus fulfilling the criteria of tumor initiating cells (TICs).

### TICs resist to high concentrations of temozolomide

Temozolomide, an oral alkylating agent, is the main drug used for high grade gliomas. The therapeutic concentration given to patients is around 60 μM. Cells cultured from tumors TG1 at three different passages, TG10 and TG16 were exposed for 48h to different concentrations of temozolomide. Cells from all cultures were highly resistant to temozolomide, 60% of the cells being still viable in presence of 1000 μM temozolomide (Table 6). No difference in temozolomide resistance was noted between long-term cultured spheres obtained from FACS-sorted CD133+ and CD133- cells. In addition, removal of growth factors for 10 days or induction of differentiation for 7 days in the presence of 10% FCS did not change TIC resistance to TMZ.

**Table 6:** TICs-derived from MGNTs are resistant to Temozolomide

	TMZ 62,5 μM	TMZ 250 μM	TMZ 500 μM	TMZ 1000 μM
**TG1 P35**	95 ± 7	94 ± 4	95 ± 5	67 ± 4

**TG1 P70**	95 ± 5	87 ± 5	83 ± 4	66 ± 6

**TG1 P90**	96 ± 5	87 ± 5	78 ± 7	58 ± 7

				

**TG10**	98 ± 6	97 ± 5	80 ± 6	59 ± 4

**TG16**	90 ± 5	80 ± 7	75 ± 5	62 ± 6

				

**TG16 CD133+**	92 ± 5	81 ± 7	74 ± 5	67 ± 8

**TG16 CD133-**	91 ± 6	75 ± 6	71 ± 5	60 ± 6

				

**TG1 without GF**	92 ± 6	85 ± 6	78 ± 6	62 ± 7

**TG1 10% SVF**	90 ± 5	80 ± 5	82 ± 6	79 ± 5

Cells cultured from tumors TG1, TG10 and TG16 were exposed for 48h to different concentrations of temozolomide (TMZ). The analysis was performed on three passages of TG1 (weeks 35, 70 and 90 after clonal selection), and on TG10 and TG16 at passage 40. It was additionally performed on cultures of TG16 initiated from CD133+ (TG16 CD133+) or CD133- (TG16 CD133-) sphere-forming cells. The influence of growth factors (GF) or serum (10%SVF) on the cells response to temozolomide was evaluated using cells cultured for 10 days without FGF/EGF or for 7 days in the presence of 10% fetal calf serum, prior temozolomide addition. Cell viability was determined using WST1 test. Results are presented as % of control. Mean ± sem of three independent experiments, each experiment performed in triplicates.

## Discussion

Tumor stem cells or tumor-initiating cells with stem-like properties (TICs) name a small subpopulation of tumor cells that are clonogenic and are capable of forming a tumor mass mimicking the original one. The present work reports on the characterization of tumor initiating cells from 12 cases of a newly characterized form of human adult glioma. These cells exhibit long-term stability in culture, and properties that support their capacity to establish a novel brain tumor from a few cells at least up to two years of continuous passages.

Within the array of brain tumors studied in this work, only MGNTs [20] yielded at a high frequency cells forming spheres in culture (12 of 15 tumors). In contrast, cells proliferating as spheres from glioblastomas, adult medulloblastoma and ganglioglioma were rapidly lost upon serial passages after 4 to 6 months in accordance with previous studies [3,4]. Most studies of glioblastomas have identified tumor stem cells in only half or less of the glioblastomas studied [3,4]. Our results raise the possibility that some of the glioblastomas assayed in these studies belong to the MGNT sub-class. Review of 200 glioblastomas showing that 50% of them contain cells expressing NFP and GFAP supports such a possibility (PV and CDD, unpublished observations). The presence of a few tumor cells co-expressing neuronal and glial antigens is one MGNT characteristic, which can be easily looked for [20]. In addition, our large set of cultured oligodendrogliomas (WHO grade II or III) never yielded any sphere and/or cells expressing a stem-like phenotype. In good agreement with this, no long-term culture from human oligodendroglioma has yet been reported. Determination of the presence or absence of tumor stem cells is, however, insufficient in the present state of knowledge to conclude that MGNTs, medulloblastomas and some glioblastomas result from the targeting of transforming mutations to an early progenitor/stem-like cell, whereas oligodendrogliomas result from mutations accumulating in a more differentiated cell.

CD133 expression, because expressed also by normal neural stem cells, has been proposed to identify cells at the top of the hierarchy formed by tumor stem cells and their more differentiated progeny, and to be therefore the paramount of glioma stem cells. Its pertinence as a glioma stem cell marker is now highly controversial, several groups having demonstrated the tumor initiating properties of CD133- cells [27]. In our hands, CD133 was rapidly down-regulated in cell spheres grown in vitro, whereas expression of other stem cell markers such as CD15 or CD34 was maintained. CD133- cells were also able to form floating cellular spheres with properties undistinguishable from those of CD133+ cells. Similarly, long-term cultured sphere forming cells, whatever established from CD133+ or CD133- tumor cells were equally resistant to temozolomide. CD133 expression most likely reflects the bioenergetics stress of the cells rather than their stem-like properties [28]. CD15, also known as SSEA1 or Lewis X, has also been recently proposed as an enrichment marker of glioma TICs [29]. Although we observed some differences in sphere sizes and proliferation rates between CD15 positive and negative cells during the first week after sorting, these variations disappeared with obtaining the secondary spheres, which in both cases contained a mixture of CD15+ and CD15- cells.

## Conclusions

We previously reported that MGNTs represent a clinical entity with distinct clinical, anatomo-pathological and radiological behaviors. We now show that they have also a distinct biological behavior among high-grade glial tumors. The glioma initiating cells described here establish a novel model that may be used to routinely establish adult human tumoral cell lines stable in long-term cultures in a define medium in a reproducible fashion. This may open new ways to identify novel tumor cell markers and surface receptor profiles for therapeutic and diagnostic purposes and to develop patient-tailored pharmacologic approaches for the cure of gliomas.

## List of abbreviations used

TIC: tumor initiating cells; MGNT: Malignant glio-neuronal tumors

## Competing interests

The authors declare that they have no competing interests.

## Authors' contributions

CP and LR carried out the cell cultures, ICC and IHC, cell sorting, proteomic studies, and drafted the manuscript. PV, NL and CDD carried out the neuropathological analysis of tumor samples and xenografts. ER and JC carried out the xenografts. AB carried out chromosomal analysis. LC participated in the FACS analysis. IB, CT, MM and HC participated in Q-PCR analysis. FRM, CT and MMM carried out the pharmacological experiments. MPJ, VMN and JH participated in the design of the study, and MPJ to redaction of he manuscript. HC conceived of the study, and participated in its design and coordination and drafted the manuscript. All authors read and approved the final manuscript.

## Pre-publication history

The pre-publication history for this paper can be accessed here:

http://www.biomedcentral.com/1471-2407/10/66/prepub

## Supplementary Material

Additional file 1 **Maintenance of tumoral architecture and cell viability in organotypic cultures of human brain tumors**. Immunohistochemical staining of an original MGNT tumor (upper panels) and after 4 weeks in organotypic culture (lower panels). The tumor samples were collected by an anatomopathologist in the surgical room, sliced into small fragments (less than 1 mm^3^), and layered on surgical sponge fragments floating over a culture medium composed of RPMI with 6% FCS. These organotypic cultures were maintained up to 6 weeks without visible alteration of the tumoral architecture, as compared to the original histological examination. Click here for file
