# Supplementary figures and images for: CD133, CD15/SSEA-1, CD34 or side populations do not resume tumor-initiating properties of long-term cultured cancer stem cells from human malignant glio-neuronal tumors
Source: BMC Cancer. 2010 Feb 24;10:66. doi: 10.1186/1471-2407-10-66 (PMC2841664; doi:10.1186/1471-2407-10-66)

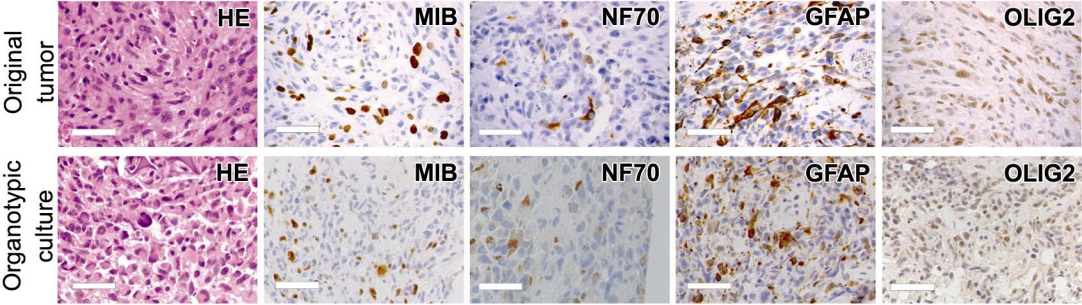

Supplementary Figure 1

Supplement: Additional file 1 — Maintenance of tumoral architecture and cell viability in organotypic cultures of human brain tumors. Immunohistochemical staining of an original MGNT tumor (upper panels) and after 4 weeks in organotypic culture (lower panels). The tumor samples were collected by an anatomopathologist in the surgical room, sliced into small fragments (less than 1 mm3), and layered on surgical sponge fragments floating over a culture medium composed of RPMI with 6% FCS. These organotypic cultures were maintained up to 6 weeks without visible alteration of the tumoral architecture, as compared to the original histological examination. [file 1471-2407-10-66-S1.PDF]
